# Supplementary figures and images for: Risk factors for toxoplasmosis in people living with HIV in the Asia-Pacific region
Source: PLoS One. 2024 Jul 1;19(7):e0306245. doi: 10.1371/journal.pone.0306245 (PMC11216616; doi:10.1371/journal.pone.0306245)

**Supplementary 1. Year of toxoplasmosis diagnosis**


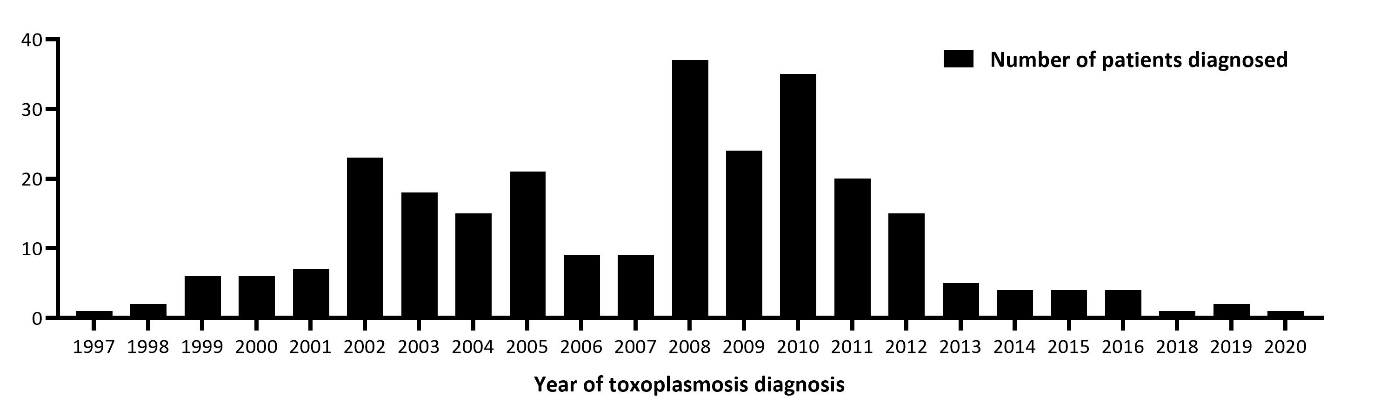

Supplement: S1 Fig — (DOCX) [file pone.0306245.s001.docx]
