# Supplementary material for: Risk factors for toxoplasmosis in people living with HIV in the Asia-Pacific region
Source: PLoS One. 2024 Jul 1;19(7):e0306245. doi: 10.1371/journal.pone.0306245 (PMC11216616; doi:10.1371/journal.pone.0306245)
Supplement: Attachment — Submitted filename: Response to Reviewers.docx [file pone.0306245.s002.docx]

[November 28, 2023]

Submission ID: PONE-D-23-29849

Title: Risk factors for toxoplasmosis in people living with HIV in the Asia-Pacific region

Dear Editor,

We appreciate the time and effort spent by you and the reviewers for providing insightful feedback to strengthen our study and improve the quality of our paper. We have made the necessary changes following the reviewer’s suggestions; the changes in the main manuscript are shown using the track changes option of MS-Word. A detailed description of our responses (red) and the changes are outlined below.

We believe that these changes have satisfactorily addressed the concerns raised, and the manuscript is now suitable for publication in the *PLOS One*.

We wish to move the manuscript closer to publication and look forward to hearing from you.

Sincerely,

Jun Yong Choi

Division of Infectious Diseases, Department of Internal Medicine

Yonsei University College of Medicine, Yonsei University Health System

50-1, Yonsei-ro, Seodaemun-gu, Seoul 03722, South Korea

Tel: +82-2-2228-1974

Fax: +82-2-393-6884

E-mail: [SERAN@yuhs.ac](mailto:SERAN@yuhs.ac)

**Response to Reviewers’ comments**

**Reviewer #1**

1. **Because patient geographical distribution and infection period difference are also critical for toxoplasmosis infection, the manuscript needs to analyze these data.**

***Author’s response #1***

We agree with the reviewer that geographical distribution and infection period differences are important in toxoplasmosis infection. Like the reviewer, we are aware of the importance of these two factors, and therefore have designed our study to analyse the site and time effect by matching by site and time period of toxoplasmosis infection so that cases and controls were compared within the same site and in a similar time frame. Matching by site and time period removed the known confounding effects of the two risk factors and allowed for comparison of cases and controls without the influence of these factors. Although matching does not output the effect sizes in the regression table for site and time period of infection (unlike the regular unmatched analyses), the regression model had “analysed” or taken into account these two factors by way of matching through the use of conditional logistic regression “conditioned on matched pairs”. Therefore, site and time were analysed by conditional logistic regression through matching. Unfortunately, we are not able to provide the number of cases by individual site to maintain confidentiality for the network, however we have listed the countries that were included, and have also further clarified the matching in our methods.

“*To account for differences in site and time period, prospective cases were matched to prospective controls, while retrospective cases were matched to retrospective controls from the same site and time period.*”

*“Factors associated with toxoplasmosis were analyzed using conditional logistic regression to account for matching.”*

*“A total of 269/9576 (2.8%) PLWH were diagnosed with toxoplasmosis from a subset of 19 TAHOD sites from Cambodia, China, Hong Kong SAR, India, Indonesia, Japan, South Korea, Malaysia, the Philippines, Singapore Taiwan, Thailand, and Vietnam, and were matched to 538 controls.”*

2. **Because many patients have not taken ART and lack CD4 & viral load data, these data deficiencies could interfere with the risk factors assessment.**

***Author’s response #2***

Thank you for your comment. As you said, it is disappointing that many of the subjects did not have CD4 or VL values. But while revising the paper this time, we excluded people without CD4 values, and found the vast majority (93.2%, instead of the previous 71%) were advanced HIV with a CD4 count of less than 200. Like your good opinion, most of the people who actually participated in the study lacked of data, but I think these results will be valuable.

3. **The IVDU etiology and HBV infection should be considered as counfounding factors and these factors are highly associated to socioeconomic status which could be the risk factor of toxoplasmosis infection.**

***Author’s response #3***

We would like to thank the reviewer for this suggestion. Injecting drug users (IDU) are normally known to be associated with hepatitis C co-infection, rather than hepatitis B. From our analysis, hepatitis C was not significant in the multivariate analysis. We have also tested for collinearity between mode of HIV exposure and HBV and found that there was no collinearity between the two variables (mean VIF=1.00).

4. **The disease type of toxoplasmosis should be described in this manuscript.**

***Author’s response #4***

We have updated the Materials and Methods section as follows; (page 5, line 133)

**Definition of toxoplasmosis**

We defined meaningful toxoplasma infection as definitive and presumptive toxoplasmosis, and definitive toxoplasmosis means patients who confirmed toxoplasmosis by microscopy (histology or cytology). Presumptive toxoplasmosis means patients whom suspicious toxoplasmosis of brain satisfying all three of the following. (i) Recent onset of a focal neurological abnormality consistent with intracranial disease or a reduced level of consciousness; (ii) Evidence by brain imaging (computed tomography or nuclear magnetic resonance) of a lesion having a mass effect or the radiographical appearance of which is enhanced by injection of contrast medium or consistent symptomatic presentation; (iii) Serum antibody to toxoplasmosis or successful response to therapy for toxoplasmosis.

**Reviewer #2**

1. **Despite in the Methods section they comment that “controls without VL or CD4 unavailable within the matched timeframe were not selected because they were considered key covariates”, 62.5% and 83.8% of controls were not tested for CD4 or VL respectively (compared with 23.4% and 70.3% in cases). Could the authors clarify this relevant aspect of their study?**

***Author’s response #1***

We apologize if this was unclear. We excluded controls without both CD4 AND VL, but allowed controls to be included if they had either a CD4 or a VL available, in order to maximize our sample size. We have clarified this in our methods:

*“The controls were randomly selected and were required to have at least a CD4 or VL available within the matched timeframe as they were considered key covariates in the study. Controls without both viral load and CD4 available were not selected.”*

2. **In the abstract they say that 71% of toxo pts had CD4 < 200 , that means that 29% had >200, what is surprising as almost all PLWH with toxo have CD4 < 200 and mostly <100. But in fact, as 23.4% of these cases were not tested for CD4, only 14/206 (7.6%) with tested CD4 had CD4 >200, thus about 92% of tested pts, had CD4 <200. Thus, the sentence in the abstract should be modified, and it should be also mentioned in the Results and Discussion sections.**

***Author’s response #2***

We would like to thank the reviewer for this suggestion. As you mentioned, I agreed to calculate the proportion of people whose CD4 values were measured, so we revised the abstract and result sections of the main text as follows, and additionally mentioned this in the discussion, as follows; (page 12, line 223)

*“Excluding 63 out of 269 people without CD4 values, 192 (93.2%) had CD4 ≤200 cells/µL and 162 (78.6%) had CD4 ≤100 cells/µL.”*

*“Furthermore, CD4 value was lower in the toxoplasmosis group compared to the control. Excluding cases without CD4 test results, the majority had advanced HIV, with 93.2% having CD4 ≤200 cells/µL. Assuming the same seroprevalence as site matching was performed, low CD4 count is a prominent risk factor for overt toxoplasma infection. Therefore, immune reconstitution through early diagnosis and use of ART remains an important task.”*

3. **It is well known that cotrimoxazole protects against toxo in immunosuppressed PLWH. The fact that in this study it seems that no differences in cotrimoxazole use was found between cases and controls, suggests that adherence to prophylaxis might be an issue in this population. Authors should comment this aspect in the Discussion section.**

***Author’s response #3***

Thank you for your comments. In fact, although the CD4 count was lower in the case group compared to the control, it was not significantly higher in those who took cotrimoxazole for prophylactic purposes. In fact, the overall cotrimoxazole administration before and after diagnosis was high in the case group. But there was no difference when analyzed with preventive purposes and it means that there may have been a problem with adherence, and this is an important result, so details about this have been added in the discussion section as follows; (page 12, line 243)

*“Also, prophylactic cotrimoxazole administration did not contribute to lowering the risk of toxoplasmosis. When we first analyzed cotrimoxazole administration before and after the diagnosis of toxoplasmosis, cotrimoxazole administration was significantly high in the case group. But when limited to prophylactic use, there was no difference from the control group. This suggests that there might be a problem with medication compliance, but our study cannot prove it.”*

4. **In the multivariate analysis, variables have been adjusted for CD4 count? It may be that PLWH being IDU or HBV+ had lower CD4 count (as probably occurred with pts not receiving ART) and if adjusting results may vary.**

***Author’s response #4***

The multivariate results were adjusted for CD4.

5. **I would say immune reconstitution instead of immune reconstruction**

***Author’s response #5***

As suggested, we have modified the pointed words in Conclusion section.
