# Supplementary material for: Risk factors for toxoplasmosis in people living with HIV in the Asia-Pacific region
Source: PLoS One. 2024 Jul 1;19(7):e0306245. doi: 10.1371/journal.pone.0306245 (PMC11216616; doi:10.1371/journal.pone.0306245)
Supplement: Attachment — Submitted filename: Response to Reviewers.docx [file pone.0306245.s003.docx]

[May 28, 2024]

Submission ID: PONE-D-23-29849

Title: Risk factors for toxoplasmosis in people living with HIV in the Asia-Pacific region

Dear Editor,

We appreciate the time and effort spent by you and the reviewers for providing insightful feedback to strengthen our study and improve the quality of our paper. We added additional data to strengthen our study. We believe that these efforts have satisfactorily addressed the concerns raised, and the manuscript is now suitable for publication in the *PLOS One*.

We wish to move the manuscript closer to publication and look forward to hearing from you.

Sincerely,

Jun Yong Choi

Division of Infectious Diseases, Department of Internal Medicine

Yonsei University College of Medicine, Yonsei University Health System

50-1, Yonsei-ro, Seodaemun-gu, Seoul 03722, South Korea

Tel: +82-2-2228-1974

Fax: +82-2-393-6884

E-mail: [SERAN@yuhs.ac](mailto:SERAN@yuhs.ac)

**Response to Reviewers’ comments**

**Reviewer #1**

1. **Thanks for your responses to the questions, and then it makes the information from the manuscript more clear. However, the case distribution and diagnosis time period are also interesting for the readers. Although these factors were matched for the analysis. I recommend that you can provide information about the case's geographical and time period distribution in a small table or in the supplement material. It is much better for the reference of our readers.**

***Author’s response #1***

We added the information about [Year of toxoplasmosis diagnosis] as supplementary 1 to the first paragraph of the “Demographics and clinical characteristics of the study population” of Result section.

**Reviewer #3**

1. **Dear Author, please: Use keywords based on MESH. Mention the prevalence and risk factors in the title. Many sources are old. Please use new sources from the last 5 years**

***Author’s response #1***

We modified the keywords to be MESH-based, and since prevalence cannot be inferred from our study, only risk factors are mentioned in the title. Lastly, we made some revisions referenced in this study.
